# Exploring Human–AI Dynamics in Enhancing Workplace Health and Safety: A Narrative Review

**DOI:** 10.3390/ijerph22020199

**Published:** 2025-01-30

**Authors:** Jakub Fiegler-Rudol, Karolina Lau, Alina Mroczek, Janusz Kasperczyk

**Affiliations:** 1 Student Scientific Society at the Department of Environmental Medicine and Epidemiology, Faculty of Medical Sciences in Zabrze, Medical University of Silesia, 41-800 Katowice, Poland; 2 Department of Environmental Medicine and Epidemiology, Faculty of Medical Sciences in Zabrze, Medical University of Silesia, 41-800 Katowice, Poland; karolina.lau@sum.edu.pl (K.L.); amroczek@sum.edu.pl (A.M.); jkasperczyk@sum.edu.pl (J.K.)

**Keywords:** occupational health and safety, artificial intelligence, human–AI interaction, workplace ergonomics, predictive analytics, worker autonomy, ethical frameworks, wearable technology

## Abstract

Background: Artificial intelligence (AI) is revolutionizing occupational health and safety (OHS) by addressing workplace hazards and enhancing employee well-being. This review explores the broader context of increasing automation and digitalization, focusing on the role of human–AI interaction in improving workplace health, safety, and productivity while considering associated challenges. Methods: A narrative review methodology was employed, involving a comprehensive literature search in PubMed, Embase, and Scopus for studies published within the last 25 years. After screening for relevance and eligibility, a total of 52 articles were included in the final analysis. These publications examined various AI applications in OHS, such as wearable technologies, predictive analytics, and ergonomic tools, with a focus on their contributions and limitations. Results: Key findings demonstrate that AI enhances hazard detection, enables real-time monitoring, and improves training through immersive simulations, significantly contributing to safer and more efficient workplaces. However, challenges such as data privacy concerns, algorithmic biases, and reduced worker autonomy were identified as significant barriers to broader AI adoption in OHS. Conclusions: AI holds great promise in transforming OHS practices, but its integration requires ethical frameworks and human-centric collaboration models to ensure transparency, equity, and worker empowerment. Addressing these challenges will allow workplaces to harness the full potential of AI in creating safer, healthier, and more sustainable environments.

## 1. Introduction

### 1.1. Defining Artificial Intelligence and Its Relevance to OHS

Artificial intelligence (AI) generally refers to computer systems designed to perform tasks that typically require human intelligence, such as learning from data (machine learning), recognizing patterns (computer vision and clustering), and making reasoned decisions (expert systems). Machine learning and deep learning algorithms, for instance, can extract insights from large datasets, thereby automating tasks and enabling predictive analytics that can improve organizational efficiency in numerous fields, including occupational health and safety (OHS) [1,2]. The International Labor Organization defines OHS as the science of the anticipation, recognition, evaluation and control of hazards arising in or from the workplace that could impair the health and well-being of workers, taking into account the possible impact on the surrounding communities and the general environment [2]. Within OHS, AI techniques have already been used to analyze injury data, forecast potential hazards, and streamline training through interactive simulations. For example, sensors and wearable devices can collect physiological and environmental data in real time, which AI-driven models use to predict incidents or recognize early signs of fatigue [3,4,5]. Although these emerging technologies bring clear advantages, such as reduced manual workload and proactive risk detection, they also raise concerns about privacy, algorithmic bias, and worker autonomy [4,6]. Although these technologies boost safety and efficiency, they also raise concerns such as reduced job control, ethical questions, data privacy risks, and the potential for job displacement [4,5]. Studies like Germany’s DiWaBe survey illustrate the mixed effects of automation on workplace dynamics: while it can enhance performance and lessen physical burdens, it may lower perceived autonomy and heighten mental fatigue [6,7]. AI systems that handle repetitive tasks often raise job satisfaction, but if they interfere with decision-making, workers can feel a loss of control and added stress [3,4,5,6,7]. To address these issues, frameworks like Parasuraman’s Levels of Automation and Kaber and Endsley’s models recommend balancing human oversight with AI support, ensuring that technology assists rather than dominates [8,9]. Such a responsible integration of AI can improve safety, productivity, and job satisfaction [10]. Nonetheless, for small and medium-sized enterprises (SMEs), the costs of specialized hardware, software, and employee training can be significant, making AI adoption challenging [4,5]. Careful planning, phased adoption, and possible financial support mechanisms can help SMEs overcome budget constraints, paving the way for human-centered AI solutions that prioritize worker autonomy, transparency, and reliability, ultimately facilitating safer and more efficient operations [1,2,3,4,5,6,7,8,9,10].

### 1.2. Legislative Framework for AI Technologies

The increasing penetration of AI in workplaces underscores the importance of legislative and regulatory frameworks that ensure safe and ethical deployment [11]. In the European Union, the proposed AI Act aims to regulate AI systems based on their risk level, ensuring that high-risk applications, including those monitoring worker behavior, are developed and used responsibly [12,13]. Other EU regulations, like the General Data Protection Regulation (GDPR), set strict standards for data privacy and consent [12]. National authorities, such as Poland’s Personal Data Protection Office (UODO) [13], further delineate workplace monitoring boundaries to safeguard employees’ personal information. Outside Europe, the United States, China, and other industrialized nations are also enacting or updating AI-specific policies, although approaches vary substantially [4,5]. In occupational safety contexts, these legislative measures seek to balance innovation—for instance, using AI to identify risks in real time—and worker protection from intrusive or discriminatory practices [14]. While the legal landscape continues to evolve, alignment between technological capability and ethical–legal requirements remains a central challenge for AI-driven OHS solutions [11,12,13,14].

### 1.3. AI for Accident Data Clustering and Risk Profiling

Beyond real-time monitoring, AI has also been applied to historical accident databases, enabling new forms of risk profiling [15,16,17]. Techniques such as unsupervised machine learning (e.g., k-means, self-organizing maps) can cluster large volumes of accident data, revealing patterns and root causes that traditional methods may miss [15]. In European contexts, public accident datasets often follow the European Statistics on Accidents at Work (ESAW) model, which standardizes the variables collected (e.g., cause, location, injury severity) [15,16,17]. Recent studies demonstrate how data clustering methods can enhance OHS strategies. Lombardi et al. applied unsupervised algorithms to ESAW-based data, identifying specific high-risk operations and environmental factors in construction sites and landfill operations [16,17,18]. Their findings help practitioners target the most hazardous tasks and develop preventive interventions tailored to the risk profile. Comberti et al. combined self-organizing maps (SOM) with k-means clustering to analyze occupational accidents in the wood industry, successfully pinpointing recurrent failure modes [19]. Additionally, Chokor et al. leveraged unsupervised machine learning to analyze OSHA injury reports in Arizona, revealing hidden patterns of co-occurring hazards and guiding more effective safety strategies [20]. These data-driven approaches supplement standard OHS assessments by unearthing complex causal relationships often masked by large or unstructured datasets. Integrating them into safety management systems could enable dynamic risk profiling, helping employers and authorities allocate resources more effectively and mitigate emerging hazards before they result in serious incidents.

### 1.4. Objectives of This Review

Building on these emerging trends, the present review seeks to explore how human–AI interaction shapes occupational health and safety, focusing on key AI techniques in OHS, including wearable technologies, predictive analytics, and clustering methods for accident data; ethical, regulatory, and organizational considerations, with an emphasis on data privacy, worker autonomy, and legislative frameworks; benefits and challenges of integrating AI into workplace safety procedures, particularly within diverse industrial sectors as well as research gaps surrounding AI deployment, especially in small and medium sized enterprises.

## 2. Materials and Methods

### 2.1. Research Scope and Identification of the Research Area

This narrative review aimed to explore the integration of artificial intelligence in occupational health and safety, focusing on its impact on workplace safety, employee well-being, and organizational efficiency. Initially, we defined our central research question: “How does the integration of artificial intelligence impact workplace safety, employee health, and organizational efficiency in occupational health and safety settings?” This question guided the inclusion of articles on AI-driven tools, technologies, and frameworks pertinent to OHS.

### 2.2. Literature Search Strategy

A comprehensive literature search was conducted in PubMed, Embase, and Scopus. We used both MeSH terms and free-text keywords to capture a wide range of relevant studies. Table 1 details the main search terms. For consistency, we opted to include studies published in the last 27 years (i.e., from 2009 to present). However, a small number of articles older than 15 years were also considered if they presented foundational theoretical models or historical frameworks relevant to human–AI interaction (*n* = 6 references).

### 2.3. Screening and Selection Criteria

A total of 654 records were identified through database searches. After removing duplicates, the remaining studies underwent title and abstract screening based on the inclusion and exclusion criteria listed in Table 2. Studies were included if they (1) addressed AI-driven strategies for identifying or mitigating workplace hazards, (2) provided outcomes related to health, safety, or organizational metrics, and (3) were peer-reviewed publications available in full text. Studies were excluded if they (1) lacked an OHS focus, (2) did not report specific findings on AI interventions, or (3) were conference abstracts, editorials, or opinion pieces without empirical or theoretical depth.

### 2.4. Full-Text Review and Data Extraction

Articles deemed eligible after the initial screening were retrieved for full-text analysis by two independent reviewers (J.F.-R. and K.L.). In cases of disagreement, a third reviewer (A.M.) resolved discrepancies. We extracted data on study design, participants or industrial sector, AI methodology or application, OHS outcomes measured (e.g., injury rates, ergonomic assessments, exposure levels), and notable limitations such as data privacy or algorithmic bias.

### 2.5. Elaboration of Results and Synthesis

**Figure 1:**
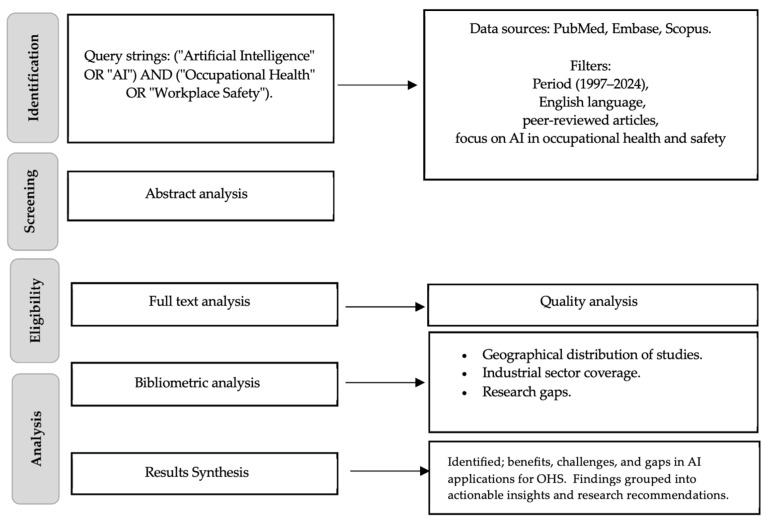
Research methodology.

In line with a narrative review approach, we summarized key findings qualitatively and grouped them thematically around (1) AI applications in wearable technology and real-time monitoring, (2) predictive analytics for risk assessment, (3) ergonomic interventions, and (4) ethical and regulatory considerations. We synthesized the evidence to identify benefits, challenges, and critical research gaps. Due to the heterogeneity of study designs and outcomes, no formal meta-analysis was performed. A summary of the methodological phases and the strategies implemented to achieve the results is presented in Figure 1.

## 3. Results

### 3.1. Overview of Included Publications

**Figure 2:**
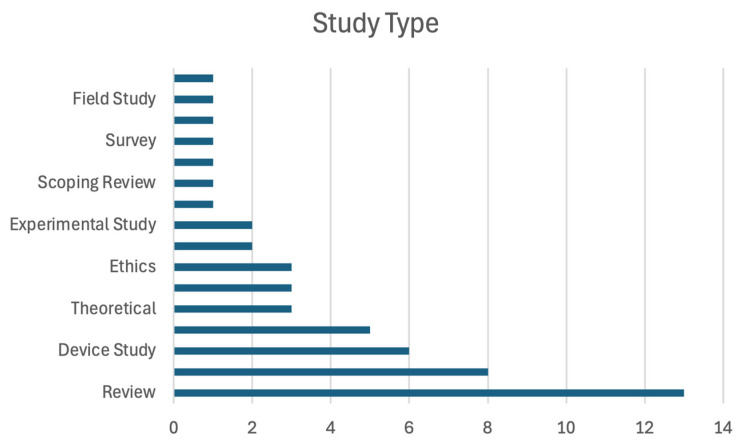
Classification of studies by type.

A total of 52 articles fulfilled the inclusion criteria and were included in this narrative review. Three guideline articles were incorporated during the peer-review process. Other studies cited in the references section were used for background information, and we did not form our main conclusions from them. Publication years ranged from 1997 to 2024, with over two-thirds of the studies appearing after 2016—a trend underscoring growing interest in AI applications in Occupational Health and Safety (OHS). Thirty-one were original research articles (e.g., observational cohort or experimental studies), 10 were review articles summarizing AI’s roles in OHS or related domains (e.g., wearable technologies, predictive analytics), and five presented theoretical or conceptual frameworks guiding the ethical and ergonomic integration of AI in workplaces. Figure 2 groups the studies by type.

**Figure 3:**
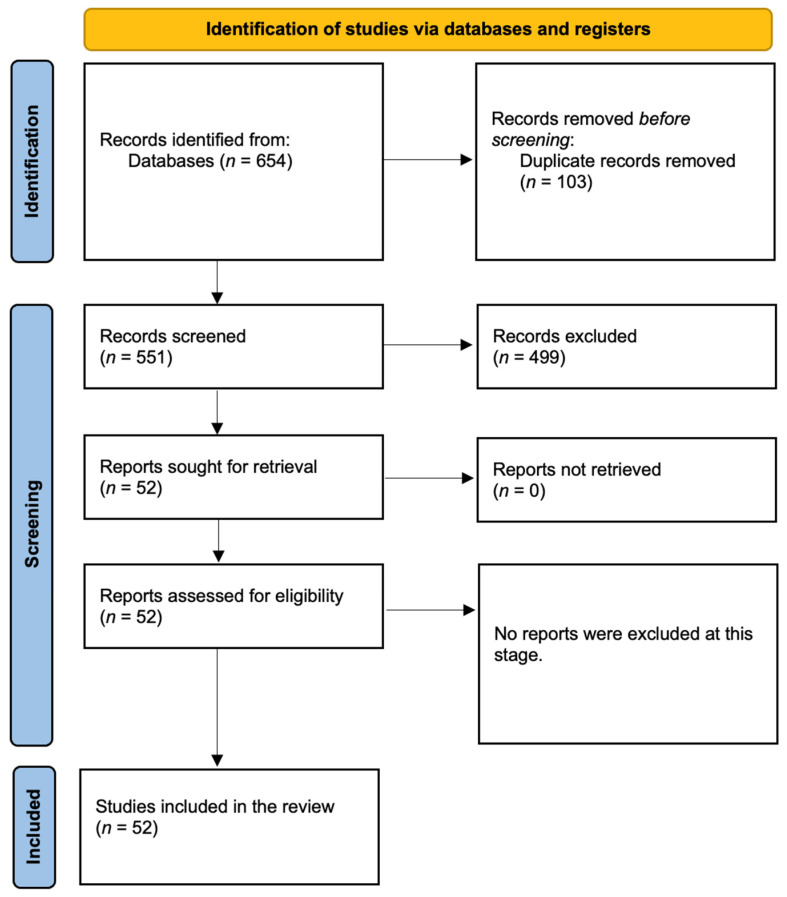
Process flow that demonstrates the research analysis in this study.

Figure 3 shows the number of results at each stage. Initial searches identified a total of 654 articles, which was further narrowed down to 551 after removing 103 duplicates. 499 records were excluded as they did not meet the inclusion criteria outlined in Table 2. No studies were excluded after this stage.

### 3.2. Bibliometric Statistics

**Figure 4:**
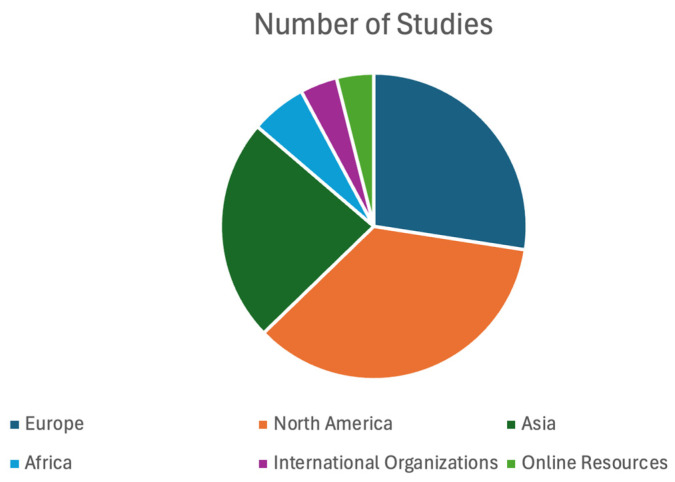
Geographical origin of papers.

In terms of geographical distribution, 14 studies originated in or were led by institutions in Europe, including countries such as Germany, Italy, Spain, Sweden, and Poland, and overarching European Union initiatives. Eighteen studies were conducted across North America, primarily the United States and Canada. Twelve emerged from Asian research settings, including countries like India, South Korea, and Iran, and regions in Southeast Asia. Three studies were from Africa, covering countries like Nigeria and Kenya. Additionally, two references pertained to international organizations, and two were categorized under online resources. Regarding workplace settings, 20 articles explored AI applications in industrial or construction environments, 10 examined healthcare settings, six focused on logistics and warehousing, while five addressed agriculture or service industries. Notably, approximately 15 publications investigated small and medium-sized enterprises (SMEs), highlighting cost and scalability concerns for AI adoption in more resource-limited contexts. Studies are grouped by country of origin in Figure 4.

### 3.3. Research Gaps

**Figure 5:**
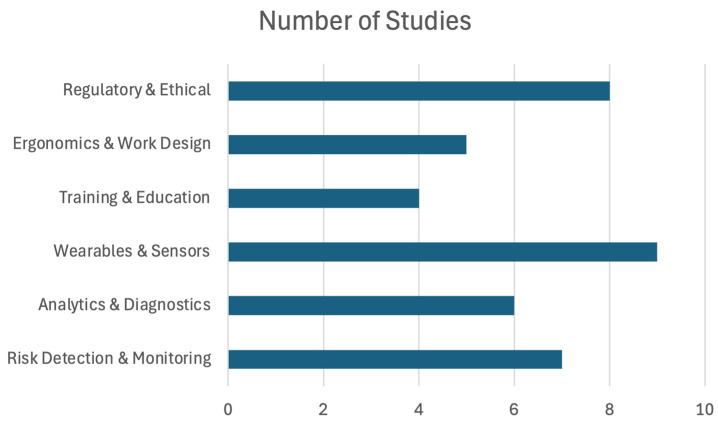
Publications categorized by major themes/topics.

Few studies have tracked long-term worker outcomes, including sustained stress levels, mental health, and technology acceptance, beyond short pilot phases. Additionally, resource constraints and limited digital infrastructure in small and medium-sized enterprises underscore the need for scalable, cost-effective AI solutions and possible policy incentives. Although privacy concerns and AI-related discrimination are frequently mentioned, concrete guidelines for everyday workplace AI governance remain underdeveloped. Furthermore, variations in metrics, methodologies, and definitions of safety outcomes limit cross-industry comparisons and hamper the generalizability of AI’s effectiveness. Moreover, the complex relationship between automation, job control, and psychological well-being calls for more human-centric designs to maintain worker autonomy and acceptance. The number of studies in each theme is presented in Figure 5.

## 4. Discussion

### 4.1. AI in OHS—Current Landscape

AI-enabled wearables, including smartwatches, biometric wristbands, and electronic textiles, enhance OHS by collecting physiological and environmental data to monitor vital signs, detect fatigue, analyze posture, and track locations in hazardous areas [21]. Industries such as construction, manufacturing, logistics, and healthcare leverage these technologies to improve ergonomics, optimize workflows, and deliver real-time hazard alerts while supporting stress monitoring, fatigue detection, and chronic condition management [22]. Advanced AI systems, such as sensors assessing air quality, noise, and employee movements, enable predictive maintenance, hazard prevention, and compliance through real-time and historical data analytics [23]. Despite these advancements, challenges remain, including data privacy, device interoperability, and user adoption, requiring secure and user-friendly AI systems [24]. As AI transforms OHS with proactive risk management and precision-driven safety measures, professionals must navigate its complexities to maximize benefits and minimize unintended impacts, fostering safer, more efficient, and worker-centered workplaces [24]. Wearable monitoring technologies provide real-time data on workers’ health and safety through devices like motion sensors (e.g., inertial measurement units, accelerometers, gyroscopes) and physiological sensors (e.g., heart-rate monitors, electrodermal activity sensors, skin temperature sensors, eye trackers, and brainwave monitors) [22,23,24]. Motion sensors capture kinematic data such as movement patterns, near-miss falls, posture, and gait, aiding in fall prevention and musculoskeletal disorder mitigation [22,23,24,25,26]. Physiological sensors measure parameters like heart rate, variability, stress, and fatigue levels through Electrocardiogram (ECG), Photoplethysmography (PPG), and Electrodermal Activity (EDA), while eye trackers assess hazard recognition and electroencephalogram (EEG) monitors mental status, including stress and cognitive load [24,25,26,27,28,29,30,31].

### 4.2. Benefits of Human–AI Interaction in OHS

Human–AI interaction in OHS provides many advantages, significantly enhancing workplace health, safety, and productivity, as demonstrated by various AI-driven solutions. One key benefit is proactive hazard identification, where AI-powered tools analyze data to detect potential risks before incidents occur, thereby preventing accidents [32]. This is illustrated by the Estimation and Assessment of Substance Exposure (EASE) software Version 2.0, August 1997 developed by the UK’s Health and Safety Executive, which uses AI to calculate exposure levels to hazardous substances across diverse work environments, enabling more precise risk management [33,34]. Another advantage is enhanced monitoring achieved through wearable devices that track key health metrics (e.g., fatigue, stress, and exposure to harmful conditions), thus enabling real-time interventions to mitigate risks [35]. Training and compliance efficiency are boosted with virtual reality and AI-powered simulations that provide immersive, effective OHS training experiences [36].

AI-assisted ergonomic evaluations help prevent musculoskeletal disorders by analyzing workplace layouts and worker movements to optimize ergonomics [37]. For instance, Lind et al. demonstrated that a wearable haptic feedback system for work technique training can significantly reduce adverse upper-arm postures and is perceived as both effective and user-friendly [38]. Furthermore, Ocharo et al. present a YOLO-based Deep Reinforcement Learning system deployed on an IoT platform integrating BLE tags, GPS modules, Fitbit devices, cameras, and CNN-based image recognition, which continuously monitors PPE usage on construction sites [39]. In parallel, Romanssini et al. found that integrating vibration-based monitoring with robust signal processing methods is crucial for predictive maintenance in rotating machinery, minimizing downtime and reducing costs [40]. AI-enhanced personal protective equipment, such as smart helmets and adaptive gear, dynamically adjusts to changing environmental conditions, ensuring continuous worker protection [41]. Finally, AI-driven drones in construction projects enable automated defect detection, predictive analytics, and real-time flight-path optimization, enhancing both safety and overall project outcomes [42].

### 4.3. Challenges, Ethical Considerations, and Summary

Artificial intelligence in occupational health introduces several ethical and practical challenges that must be addressed to ensure its responsible integration. A primary concern is data privacy and security, as the monitoring of employees’ behavior and health information raises questions about protecting sensitive data [11,12]. In the European Union, the General Data Protection Regulation (GDPR) imposes strict requirements for handling personal data, including obtaining valid consent and implementing robust safeguards [11]. Poland’s labor laws also set specific rules for monitoring employees, overseen by national authorities like the Personal Data Protection Office (UODO) [12]. Additionally, upcoming EU legislation (e.g., the proposed AI Act) may further refine regulations for AI-driven workplace practices [13]. The Vatican City has also issued guidelines emphasizing the ethical use of artificial intelligence, grounded in principles such as transparency, accountability, and respect for human dignity [43]. These guidelines advocate for AI systems to prioritize human well-being, ensure fairness, and prevent harm, reinforcing the moral responsibilities of both developers and users of AI technology [16]. Alongside these legal frameworks, workforce mental health can be affected by anxiety over AI surveillance and potential job losses [34]. Bias in AI systems is also a key concern, where discriminatory algorithms could unfairly influence performance evaluations or hiring, aggravating workplace inequalities [14]. High costs and uneven distribution of AI tools—especially for SMEs—contribute to inequitable adoption [15].

### 4.4. Human–AI Collaboration Models

The Human-in-Control model in human–AI collaboration builds upon frameworks like Parasuraman’s Levels of Automation, which categorizes automation based on the degree of human involvement, and Kaber and Endsley’s models, emphasizing situational awareness and human decision-making in complex systems [8,9,10,40]. For example, in OHS, AI can autonomously monitor worker fatigue and stress (lower automation), but the final decision to intervene remains with supervisors to ensure contextual appropriateness [40,41,42,43]. Additionally, theories like Ulrich’s resource-based view and the Job Demand-Control (JDC) model highlight how AI can reduce workloads while still requiring humans to manage cognitive and ethical oversights, preserving both performance and well-being [44]. The DiWaBe survey in Germany underscores that while digitalization and AI can improve efficiency and reduce physical workloads, they may increase job demands, workplace stress, and the need for upskilling, reinforcing the importance of balanced technology integration [6,10]. Recent literature also showcases the role of data clustering in large-scale accident databases, aimed at identifying specific risk profiles [20,21,22,23,24,25,26,27,28,29,30,31,32,33,34,35,36,37,38,39,40]. By grouping accidents that share similar attributes, clustering algorithms can uncover hidden patterns and high-risk scenarios, thereby improving predictive modeling [44,45]. In OHS contexts, such data-driven insights should always be paired with expert human oversight: professionals and supervisors can interpret cluster outputs, validate them against real-world conditions, and adapt safety strategies accordingly [44,45]. This human–AI synergy ensures that preventive measures are both evidence-based and grounded in workplace realities.

### 4.5. Examples of Human–AI Collaboration

Human–AI cooperation in OHS is becoming more common in countries with strong industrial sectors and robust research ecosystems—such as the United States, Germany, Japan, and select EU member states—where AI applications have moved from experimental to routine use [46,47]. In these regions, AI-powered predictive analytics, harnessing data from sensors and wearables, can detect potential hazards (e.g., worker fatigue or harmful exposure) in real time, enabling managers to adjust shifts or update safety protocols [48]. In ergonomics, AI often analyzes workplace layouts and human postures, while supervisors validate those insights and customize solutions for the specific context [49]. Surveillance tools leveraging AI also monitor machinery, detecting malfunctions early and allowing technicians to respond proactively [50]. During emergencies, AI simulations predict fire or chemical spill trajectories, enabling human experts to interpret outcomes and coordinate evacuations [51]. Although smaller organizations may still face adoption barriers, these examples highlight how AI can reinforce daily decision-making and hazard mitigation, while human expertise ensures ethical deployment, contextual understanding, and worker trust [52,53]. Table 3 summarizes the main aspects of this.

### 4.6. Future Directions

Looking ahead, OHS research will demand ethical and transparent frameworks for responsible AI integration, addressing legislative challenges and the need for comprehensive training [46,47,48]. As AI tools become embedded in daily workflows, policymakers must refine existing regulations—such as GDPR in the European Union and the proposed EU AI Act—to address algorithmic bias, data privacy, and accountability [45,47]. Governments and professional bodies should establish standards that ensure safe AI deployment without compromising equity or worker well-being [4,29,30,31,32,33,34,35,36,37,38,39,40,41,42,43,49,54]. Long-term studies are necessary to assess AI’s psychological effects on workers, including anxiety, job security concerns, and broader mental health implications, enabling organizations to implement effective mitigation strategies [29,30,31,32,33,34,35,36,37,38,39,40,41,42,43,54]. OHS researchers must also explore AI’s potential in predictive safety measures, ergonomics, and mental health monitoring, emphasizing sustainable, equitable, and worker-centric practices [43,45]. Beyond the workplace, AI literacy and human–AI collaboration skills must become integral to educational curricula [49]. By familiarizing students with advanced AI systems, schools and training programs can cultivate a workforce prepared for future OHS technologies and promote responsible innovation [4,50,51]. Ultimately, achieving these goals will require a balanced approach that combines legislation, industry-specific guidelines, and continuous education. This collective effort—uniting policymakers, educators, industry leaders, and OHS professionals—will help ensure that AI adoption enhances worker safety and autonomy without undermining equity or well-being. AI has the potential to support the achievement of Sustainable Development Goals (SDGs) 3 (Good Health and Well-being) and 8 (Decent Work and Economic Growth) outlined in Agenda 2030. Our findings emphasize the role of AI-driven occupational health and safety (OHS) in promoting sustainable workplace practices [55].

### 4.7. Limitations of the Present Study

Despite these insights, several limitations must be acknowledged. First, the temporal scope of our review may exclude recent or emerging trends, potentially affecting the generalizability of our findings. Second, our inclusion of specific types of publications and workplaces (e.g., those from predominantly industrial or construction-focused sectors) may not capture the full range of scenarios where AI can influence OHS. Third, a language bias toward English-language literature may omit significant regional findings. Finally, cross-country variations in legal, cultural, and technological adoption could introduce complexities not fully addressed here. Recognizing these parameters is crucial for properly interpreting the study’s conclusions and guiding subsequent research aimed at validating and extending our findings across diverse occupational environments.

## 5. Conclusions

AI integration in OHS can significantly reduce workplace hazards, enhance ergonomic designs, and streamline workflows. Real-time hazard detection, wearable monitors, and predictive analytics lower injury rates, promote healthier environments, and increase productivity. Machine learning enables proactive risk identification and intervention, supporting OHS professionals in optimizing resources and tailoring safety measures. However, challenges include ensuring data privacy and security, mitigating algorithmic biases, and preventing overreliance on AI, which can affect worker autonomy and increase stress. Smaller organizations may struggle with implementation, potentially widening the gap with larger firms. A human-centered approach is essential, where AI complements rather than replaces human oversight. Transparent communication, ethical guidelines, and stakeholder engagement are crucial to ensure AI-driven OHS systems enhance worker well-being and organizational efficiency responsibly. By addressing these concerns, AI can be harnessed to create safer, healthier, and more equitable workplaces.

## Figures and Tables

**Table 1:** MeSH keywords used in the study.

Category	MeSH Keywords
General Keywords	“Occupational Health”, “Workplace”, “Occupational Safety”, “Occupational Health Services”, “Occupational Exposure”
AI-Related Keywords	“Artificial Intelligence”, “Machine Learning”, “Deep Learning”, “Automation”, “Decision Support Systems, Clinical”
Health and Safety Metrics	“Ergonomics”, “Risk Assessment”, “Workplace Monitoring”, “Safety Management”, “Health Promotion”
Psychological and Social Aspects	“Stress, Psychological”, “Mental Fatigue”, “Job Satisfaction”, “Mental Health”
Applications and Tools	“Wearable Electronic Devices”, “Sensors”, “Predictive Analytics”, “User–Computer Interface”

**Table 2:** Selection criteria for papers included in this review.

Inclusion Criteria	Exclusion Criteria
Studies and real-world applications of AI in OHS. Clinical or observational studies assessing the impact of AI on workplace safety, health metrics, and decision-making. Review articles, meta-analyses, and theoretical frameworks relevant to human–AI interaction in OHS. Recent and relevant studies focusing on AI tools for improving ergonomics, monitoring hazards, and enhancing OHS outcomes. Investigations addressing psychological and social dimensions of AI integration in workplaces, such as job satisfaction, mental health, and ethical concerns. Outcomes related to improvements in workplace safety, health metrics, worker autonomy, productivity, and employee well-being. Articles published in English within the last 27 years to ensure relevance.	Studies not related to AI applications in OHS or lacking clear focus on workplace health and safety improvements. Editorials, commentaries, opinion pieces, and gray literature without empirical evidence. Studies lacking specific outcomes or mechanisms related to human–AI interaction or safety metrics. Articles with incomplete or inaccessible data, or without full-text availability. Publications in languages other than English without translation. Articles older than 27 years to maintain the relevance of findings.

OHS—occupational health and safety.

**Table 3:** Advantages and Challenges of Human–AI Collaboration in OHS.

Dimension	Advantages	Challenges
Risk Detection & Monitoring	Proactive hazard identification and preventionReal-time alerts & predictive maintenance [1,2,3]	Data privacy and security concernsDevice interoperability and system integration [15,16]
Analytics & Diagnostics	Early malfunction detectionAdvanced data clustering for risk profiling [4,5]	AI bias leading to unfair treatmentOverdependence on AI for critical decisions [17,18]
Wearables & Sensors	Continuous physiological monitoring (fatigue, stress)Quick intervention and more targeted prevention [6,7,8]	Worker acceptance and comfort with wearablesInteroperability and reliability of devices [19,20]
Training & Education	VR/AR simulations enhance engagementPersonalized feedback for skill improvement [9,10]	High cost for SMEsSkills gap and need for upskilling in AI literacy [21,22]
Ergonomics & Work Design	AI-driven assessment reduces musculoskeletal disordersImproved workflow efficiency [11,12]	Implementation time and resourcesPotential for job stress if changes are too rapid [23,24]
Regulatory & Ethical	Potential alignment with compliance requirements (e.g., GDPR)Improved accountability through AI-based tracking [13,14]	Regulatory uncertainty and evolving AI ActsEthical concerns regarding surveillance and algorithmic transparency [25,26]

VR: Virtual Reality; AR: Augmented Reality; AI: Artificial Intelligence; SME: Small and Medium-Sized Enterprise; GDPR: General Data Protection Regulation.

## Data Availability

Not applicable.
